# High mobility group box 1 levels in septic disseminated intravascular coagulation patients undergoing Polymixin-B immobilized fiber-direct hemoperfusion

**DOI:** 10.1186/cc10987

**Published:** 2012-03-20

**Authors:** Y Ishibe, Y Suzuki, H Sato, G Takahashi, M Kojika, N Matsumoto, Y Inoue, S Endo

**Affiliations:** 1 Iwate Medical University, Morioka, Japan

## Introduction

The serum levels of high mobility group box 1 (HMGB1) were examined in patients with septic disseminated intravascular coagulation (DIC) undergoing Polymixin-B immobilized fiber-direct hemoperfusion (PMX-DHP).

## Methods

The subjects were 16 patients with serum endotoxin levels of 1.1 pg/ml or over. The average APACHE II score was 32.2, the average SOFA score 12.4, and the average DIC score 5.5.

## Results

Following PMX-DHP, the serum endotoxin level decreased to below the limit of detection in all patients. The serum HMGB1 level decreased significantly to 31.2, 16.6 and 7.9 ng/ml on days 0, 1, and 2, respectively. The average of the DIC score improved from 5.6 to 3.9 to 2.9. Overall, the 30-day, 60-day, 90-day and 180-day mortality rates were 0, 6.3%, 12.5% and 12.5%, respectively.

## Conclusion

Following initiation of PMX-DHP, the serum HMGB1 level decreased and the DIC score also decreased accordingly.

